# Tyrosinase Inhibitory and Antioxidant Activity of Enzymatic Protein Hydrolysate from Jellyfish (*Lobonema smithii*)

**DOI:** 10.3390/foods11040615

**Published:** 2022-02-21

**Authors:** Maytamart Upata, Thanyaporn Siriwoharn, Sakunkhun Makkhun, Suthasinee Yarnpakdee, Joe M. Regenstein, Sutee Wangtueai

**Affiliations:** 1 Faculty of Agro-Industry, Chiang Mai University, Chiang Mai 50100, Thailand; deariimaytamart@hotmail.co.th (M.U.); thanyaporn.s@cmu.ac.th (T.S.); suthasinee.y@cmu.ac.th (S.Y.); 2 Faculty of Agriculture and Natural Resources, University of Phayao, Phayao 56000, Thailand; sakunkhun.ma@up.ac.th; 3 Department of Food Science, College of Agriculture and Life Science, Cornell University, Ithaca, NY 14853-7201, USA; jmr9@cornell.edu; 4 College of Maritime Studies and Management, Chiang Mai University, Samut Sakhon 74000, Thailand

**Keywords:** antioxidant activity, anti-tyrosinase activity, protein hydrolysate, jellyfish, alcalse, flavourzyme, papain, *Lobonema smithii*

## Abstract

The optimization of antioxidant and anti-tyrosinase activity during jellyfish hydrolysate preparation was studied using a response surface methodology (RSM) with a face-centered composite design. The influence of the hydrolysis duration and the enzyme concentration on the IC_50_ of the DPPH and ABTS radical scavenging activity, ferric-reducing antioxidant power (FRAP), the degree of hydrolysis (DH), yield, and the IC_50_ value of tyrosinase inhibitory activity were determined. The optimum conditions for the production of jellyfish hydrolysate using alcalase (JFAH), flavourzyme (JFFH), or papain (JFPH) were achieved at hydrolysis times of 360, 345, or 360 min, respectively, and at an enzyme concentration of 5.0%. JFFH had the highest antioxidant and tyrosinase inhibitory activity. JFAH, JFFH, and JFPH concentrations of 2.5 mg/mL resulted in HaCaT cells (IC_80_) having a survival rate of 80%. The amino acid profile of JFFH contained about 43% hydrophobic and 57% hydrophilic amino acids, comprising Gly, Cys, Glx, Asx, which were dominant. The isolation of a peptide fraction from JFFH was carried out using ultrafiltration membranes (10, 3, and 1 kDa) and gel filtration chromatography. Fraction-III (1–3 kDa) showed the highest antioxidative and tyrosinase inhibitory activity.

## 1. Introduction

Tyrosinase, a copper-containing molecule, is an essential enzyme in melanin formation that accelerates the generation of melanin from tyrosine via oxidation. The enzyme is predominantly involved in two melanin production reactions, i.e., the monophenolase reaction involving the hydroxylation of tyrosine and the diphenolase reaction for the oxidation of 3,4-dihydroxyl-l-phenylalanine (l-DOPA) to dopaquinone [1]. Melanin is a pigment that is found in bacteria, fungals, plants, and mammals. Tyrosinase activity is necessary for the provision of melanosomes into keratinocytes, and melanogenesis modulators can directly affect this activity [2]. Therefore, limiting tyrosinase activity in cosmetics and medicine can prevent the browning of foods and melanin synthesis [3]. The development and screening of tyrosinase inhibitors to be used as cosmetics products and food additives has been of interest in the industrial sector [4,5,6].

Edible jellyfish have been consumed throughout Southeast Asia for more than a thousand years [7,8]. The total world capture production of edible jellyfish in 2015–2018 was estimated to be ~300,000 tonnes/year [9]. Most commercial jellyfish are processed using a traditional method involving alum salt dehydration before producing the product as being canned in brine, semi-dried, or dry-salted [8]. White jellyfish (*Lobonema smithii*) are one of the main edible species of jellyfish in Asia. In Thailand, it is used to produce a salted jellyfish product for both domestic consumption and for export to other consumer countries, i.e., Taiwan, China, Malaysia, South Korea, and Japan, with a value about USD 10 million annually [10,11]. Jellyfish are abundant in collagenous protein, have a high nutritional value, and is capable of pharmacological activities that might imply a wider range of applications in the food, cosmetic, and pharmaceutical industries [7,12]. In China, it has been utilized for the treatment of bronchitis, high blood pressure, tracheitis, asthma, and gastric ulcers [13]. Type I collagen and type A gelatin from jellyfish have been prepared and characterized for further applications [11,14,15]. Based on the supply and potential health benefits, jellyfish may be used to develop functional ingredients for health foods, including nutraceutical products.

Protein hydrolysates are chemically or enzymatically produced and yield free amino acids or short peptides. Hydrolysis using enzymes has been utilized to obtain nutritional and bioactive peptides [16,17]. The antioxidant and tyrosinase inhibition activity of protein hydrolysates and peptides from marine resources have shown strong antioxidant activity and have been applied as natural antioxidants [18,19]. They have also been shown to be tyrosinase inhibitors with low adverse side effects [4]. Jellyfish collagen hydrolysates and peptides demonstrate antioxidant activity [7,12,20,21], melanogenesis inhibition [12], tyrosinase inhibition [21], anti-fatigue activities [7], UV protection [14], and angiotensin converting enzyme (ACE) inhibitory activity [22]. However, these peptides from white jellyfish have not been characterized, nor have preparative methods been maximized for tyrosinase inhibitory and antioxidant activities.

The aim of this research was to develop value-added products from low-value salted white jellyfish from Thailand for potential applications in the food, cosmetics, and pharmaceutical industries. Therefore, the production of jellyfish hydrolysates with tyrosinase inhibitory and antioxidant activity was optimized using enzymatic hydrolysis and peptide isolation.

## 2. Materials and Methods

### 2.1. Raw Material and Preparation

Alum-salted jellyfish (*Lobonema smithii*) was obtained from Siam Jellyfish Co., Ltd., Samut Sakhon, Thailand. It was contained in a sealed high-density polyethylene (HDPE) bucket (size 18 kg). To desalt the jellyfish, the sample was initially cleaned under running tap water and was soaked in water (jellyfish:water = 1:2) overnight (12–14 h). The soaked jellyfish was cleaned under running tap water until the salt content in wash water was approximately zero. The salt content was measured using a salinometer (Master-S28 M, Atago^®^, Tokyo, Japan), and the water was drained for 30 min to obtain the desalted jellyfish. The jellyfish were ground using a blender (Daily Collection HR7627 Blender, Philips, Bangkok, Thailand) and were packed in Zip-lock polyethylene bags and stored at −18 to −20 °C until use (<2 months).

### 2.2. Chemical and Enzymes

Papain (≥3 activity units (AU)/mg), Alcalase 2.4 L[≥2.4 (AU)/g], flavourzyme (≥500 AU/g), tyrosinase from mushroom, 1,1-diphenyl-2-picrylhydrazyl (DPPH), ferric chloride hexahydrate (FeCl_3_•6H_2_O), 2,2′azinobis(3-ethylbenzothiazoline-6-sulfonic acid) (ABTS), 2,4,5-tripyridyl-triazine (TPTZ), 2,4,6-trinitrobenzenesulfonic acid (TNBS), kojic acid, and 3,4-dihydroxy-L-phenylalanine were purchased from Sigma-Aldrich (St. Louis, MO, USA). Fetal bovine serum (FBS), penicillin, dulbecco’s modified eagle medium (DMEM), and l-glutamine were purchased from XL Biotec Co., Ltd. (Bangkok, Thailand). All of the chemicals and reagents used were of analytical grade.

### 2.3. Chemical Composition Analysis

The chemical composition of desalted jellyfish was determined according to the official AOAC method (2000): 934.01 moisture, 954.01 crude protein, 991.36 lipids, and 942.05 ash. Nitrogen was converted to crude protein using a conversion factor of 6.25.

### 2.4. Optimization of the Production of Jellyfish Hydrolysate (JFH)

The desalted jellyfish was hydrolyzed using three different proteases. The effect of the enzyme concentration (1–5% *w*/*w* protein content) and hydrolysis time (60–360 min) on the yield, DH, FRAP value, and antioxidant and tyrosinase inhibition activity were determined. For the hydrolysis process, a mixture of 50 g desalted jellyfish and 100 mL distilled water (*w*:*v =* 1:2) was terminated for endogenous enzymes by heating it at 95 °C for 15 min, cooling it to the optimum temperature of each enzyme (papain and flavozyme at 50 °C and alcalase at 60 °C), and hydrolyzing it following the experimental design. The reaction was carried out in a shaking incubator (Memmert WNB45, Schwabach, Germany) with constant agitation for 60–360 min (Table 1). After hydrolysis, the reaction of the hydrolysate solution was stopped by heating it at 95 °C for 15 min, and it was then cooled under running tab water. Thereafter, the hydrolysate solution was centrifuged at 5500× *g* for 15 min (1736R, LaboGene, Lynge, Denmark). The supernatant was lyophilized using a freeze-dryer (GRISRIANTHONG, GFD-3H, Samut Sakhon, Thailand). The jellyfish hydrolysate powder that was obtained was packed in Zip-lock polyethylene bags and kept at −20 °C until further investigation.

### 2.5. The Degree of Hydrolysis (DH) Analysis

The DH of the jellyfish hydrolysate solution after hydrolysis was determined according to the method described by Mongkonkamthorn et al. [19]. The 125 µL sample was diluted with 2 mL Na_3_PO_4_ buffer (pH 8.2), and 1 mL TNBS solution (0.01% prepared with 0.2125 M phosphate buffer pH 8.2) was added. The mixture was shaken and kept in the dark at 50 °C for 30 min. After that, 2 mL of 0.1 M Na_2_SO_3_ was added into the mixture to terminate the activity, and the mixture was cooled at 25–27 °C for 15 min, and the absorbance of the mixture was measured at 420 nm using a microplate reader (Varioskan^TM^ LUX, Thermo Scientific^TM^, Waltham, MA, USA). The α-amino group was estimated in terms of 2-Amino-4-methylpentanoic acid (l-leucine) (Sigma-Aldrich, St. Louis, MO, USA). The following equation was used to determine the DH (1).

DH (%) = [(L_H_ − L_0_)/(L_max_ − L_0_)] × 100 (1)

where L_H_ is the amount of Leu equivalence obtained from jellyfish hydrolysates. L_0_ is the amount of Leu equivalence in enzymatic hydrolysate at the initial time. L_max_ is the total amount of Leu equivalence in the initial enzymatic hydrolysate taken after hydrolysis in 6 M hydrochloric acid at 100 °C for 12 h.

### 2.6. DPPH Radical Scavenging Activity

The scavenging activity of the DPPH radical was determined using the modified method of Ahn et al. [23]. The 100 µL sample was added 100 µL of DPPH solution (0.1 mM in 70% ethanol), and the mixture was kept in the dark at 25–27 °C for 30 min. Distilled water was used as the control and as a substitute sample. An absorbance was obtained at 517 nm using the microplate reader. The scavenging activity was expressed using Equation (2):

Scavenging activity (%) = [(A_control_ − A_sample_)/A_control_] × 100 (2)

A_control_ is the absorbance of the DPPH solution. A_sample_ is the absorbance of the sample mixed with DPPH. The activity was presented as IC_50_ (sample concentration required to inhibit 50% of initial DPPH concentration). The IC_50_ value was estimated from the linear regression graph to optimize the process and µmol Trolox equivalents (TE)/g sample for the tested fractions.

### 2.7. ABTS Radical Scavenging Activity

The inhibition activity of the ABTS radical was determined in accordance with the method provided by Ahn et al. [23]. An ABTS solution (7 mM ABTS and 2.45 mM K_2_S_2_O_8_ were mixed together in a 1:1 (*v*/*v*) ratio) was prepared and allowed to activate in the dark for 16–18 h at 4 °C. Then, it was diluted with 70% ethanol to acquire an ABTS working solution (absorbance of 0.7 ± 0.05 at 734 nm). The 100 µL of the reaction mixture and 1.9 mL of the ABTS working solution was mixed and kept in the dark at 25–27 °C for 8 min. The resultant solution was obtained at 734 nm. The radical scavenging activity ABTS, and the IC_50_ value were calculated in a similar manner as in the DPPH analysis in order to optimize process and the µmol Trolox equivalents (TE)/g sample for the tested fractions.

### 2.8. Ferric Reducing Antioxidant Power (FRAP) Value

The FRAP value was measured following the method of Wangtueai et al. [24]. A fresh FRAP solution was produced by mixing of 20 mM FeCl_3_ and 6H_2_O in deionized water, 10 mM TPTZ solution in 40 mM HCl, and 300 mM acetate buffer (pH 3.6) at ratio of 1:1:10 (*v*:*v*:*v*). The FRAP solution was kept in a water bat at 37 °C for 30 min. An amount of 150 µL of the sample was mixed with 2.85 mL FRAP solution and was reacted for in the dark 30 min. The absorbance of the resultant solution was obtained at 593 nm. The FRAP values were expressed in mmol FeSO_4_/g sample. Additional dilutions were carried out if the FRAP value was at a higher range in the linear standard curve.

### 2.9. Tyrosianse Inhibitory Activity

The tyrosinase inhibition activity of jellyfish hydrolysate was measured according to a slightly modified version of the method discussed in Chan et al. [25]. A solution comprising 3.2 mM l-DOPA in 50 mM phosphate buffer (pH 6.8) was prepared. This reaction was generated in a multi-well plate. An amount of 10 µL of various concentrations of JFH was diluted with 40 µL of 50 mM K_3_PO_4_ buffer (pH 6.8) and 70 µL of tyrosinase (150 units/mL activity), and the mixture was allowed to react for 10 min. Then, 80 µL 3.2 mM L-DOPA was added to each well and incubated for another for 10 min. The absorbance of the resultant solution was measured at 475 nm using a multi-well plate reader. Kojic acid was used to as a positive control. The inhibitory activity of tyrosinase was expressed following Equation (3):

Tyrosinase inhibitory activity (%) = {[ A_control_ − (A_sample_ − A_colour_)]/(A_control_ − A_blank_)} × 100 (3)

where A_blank_ is the absorbance of deionized water. A_colour_ is an absorbance of a mixture comprising the sample with phosphate buffer. A_control_ is the absorbance of a mixture comprising buffer, tyrosinase, and L-DOPA without sample. A_sample_ is the absorbance of solution resulting from the reaction.

The IC_50_ values were calculated in a similar manner as the DPPH analysis.

### 2.10. Amino Acid Analysis

The Amino acid composition of the jellyfish hydrolysate with flavourzyme hydrolysis (JFFH) powder was analyzed using HPLC (HP 1260, Fluorescence Detector, Agilent Technologies, Waldbronn, Germany) according to Herbert et al. [26]. The standard mixture of amino acids (Sigma-Aldrich, St. Louis, MO, USA) was applied for both identification and quantitation. The amino acid content was expressed in g amino acid/100 g of JFFH.

### 2.11. In Vitro Cytotoxicity Determination

Human keratinocytes cells (HaCaT cell line) were purchased from XL Biotec Co., Ltd. (Bangkok, Thailand). HaCaT cells were plated in DMEM containing 10% FBS, 2 mM glutamine, and 1% penicillin (100 U/mL) in a 5% CO_2_ atmosphere at 37 °C. The effects of jellyfish hydrolysate on HaCaT cell cytotoxicity and cell viability were measured using the MTT value determined by Pastorino et al. [27]. Briefly, HaCaT cells were seeded on a 96-well culture plate at 1 × 10^4^ cell/well (100 µL) and were incubated 12 h to obtain cells that were attached to the substratum. The cells were treated with JFFH, JFAH, and JFPH at a various concentration (312.5, 625, 1250, 2500, and 5000 µg/mL), and some were left untreated (control) for 24 h at 37 °C. After incubation, 15 µL of the MTT solution (5 mg/mL in PBS) was added to each well. After subsequent incubation for 4 h, the purple-colored precipitates were obvious. The supernatant was removed, and the formazan precipitates were solubilized with the addition of 100 µL DMSO per well. Then, after 10 min of incubation, absorbances were obtained at 540 and 630 nm using a scanning multi-well microplate reader (SpectraMax i3x, Molecular Devices, CA, USA). The cell viability was expressed using Equation (4).

% Cell viability = [(OD of treated)/(OD of control)] × 100(4)

where OD _treated_ is OD _treated_540 − OD _treated_630, OD _control_ is OD _control_540 − OD _control_630.

### 2.12. Isolation of Antioxidant and Anti-Tyrosinase Peptides from Jellyfish Hydrolysate

#### 2.12.1. Fractionation of Jellyfish Hydrolysate by Ultrafiltration

The JFFH were fractionated using an Amicon^®^ stirred cell (Merck KGaA, Darmstadt, Germany) with an ultrafiltration membrane bioreactor system with a range of molecular weight cut-offs: 10, 3, and 1 kDa. Sample (10 g) was dissolved in deionized water (200 mL) (5% *w*:*v*) and fractionated using a nominal series of MWCO membranes: 1, 3 and 10 kDa. Four peptide fractions (fraction I (>10 kDa), fraction II (3–10 kDa), fraction III (1–3 kDa) and fraction IV (<1 kDa)) were collected and freeze dried. The JFFH fractions were analyzed to determine their antioxidant and tyrosinase inhibitory activity.

#### 2.12.2. Gel Filtration Chromatography

The JFFH fraction with the highest anti-tyrosinase and highest antioxidant activity obtained from the ultrafiltration membrane was further separated using a gel filtration column (2.6 × 70 cm) with a Sephadex G-25 (GE Healthcare Bio-Science AB, Uppsala, Sweden). The low-pressure chromatography system (BioLogic LP system, Bio-Rad, Hercules, CA, USA) with a connected fraction collector (Biofrac Fraction Collector, Bio-Rad Laboratories) was used. In brief, 100 mg sample was dissolved in 2 mL distilled water, filtered through a 0.45 µm syringe filter PTFE (F13-PT045, Chrom Tech, MN, USA), injected onto a column, eluted with distilled water at a 0.5 mL/min flow rate, and collected with 3 mL eluted solution/fraction. The absorbances of the fractions were determined at 220 and 280 nm, as were the DPPH and ABTS radical scavenging activity, FRAP value, and tyrosinase inhibitory activity. The void volume of the column was measured using blue dextran (2000 kDa). Molecular-weight (MW) protein standards (tyrosine (181 Da), Gly-Try (238 Da), vitamin B12 (1356 Da), and insulin chain B (3496 Da)) were used for peptide MW estimation.

### 2.13. Experimental Design

The optimum conditions for jellyfish hydrolysis were determined using a response surface methodology with a face-centered composite design (two-factor three-levels). The independent variable effect (the hydrolysis time (X_1_, 60–360 min) and the enzyme concentration (X_2_, 1–5%)) on the DH, yield, IC_50_ value of the DPPH and ABTS radical scavenging and tyrosinase inhibition activity (mg/mL), and FRAP value (mmol FeSO_4_/g sample) were evaluated. The experiments with each enzyme consisted of 11 treatments (8 incomplete factorials and 3 replicated central points), as shown in Table 1. The design of the experimental, analysis and response surface plots were carried out using Design Expert software (version 11, Stat-Ease, Inc., Minneapolis, MN, USA). A full quadratic model for each response was obtained and expressed with real variables using following Equation (5):

Yi *=* β_0_ + β_1X1_ + β_2_x_2_ + β_11_x_1_^2^ + β_22_x_2_^2^ + β_12_x_1_x_2_ + ε (5)

where Y_i_ is the response variables, and x_1_ and x_2_ are the independent variables, whereas ε represents the random error, and β_0_, β_1_, β_2_, β_11_, β_22,_ and β_12_ are the coefficients for the constant, linear, quadratic, and interaction terms.

One-way analysis of variance (ANOVA) was carried out using SPSS Statistical software version 17 (SPSS Inc., Chicago, IL, USA), and the means comparison (*p ≤ 0.05*) was conducted using Duncan’s new multiple range test (DMRT).

## 3. Results and Discussion

### 3.1. Chemical Composition of Jellyfish

The Chemical composition of the desalted jellyfish (rehydrated jellyfish) was 82.6% moisture, 13.1% crude protein, 4.1% ash, and 0.6% fat content. A previous report by Rodsuwan et al. [11] showed the proximate compositions of dried desalted jellyfish as being 70% protein, 10% moisture, 9% fat, and <1% ash content. The composition of the jellyfish tissue was largely determined based on the bodies of water in which these invertebrates were discovered. In dried *Stomolophus meleagris*, *Cotylorhiza tuberculate*, *Rhizostoma pulmo*, and *Lobonema smithii*, protein and ash were the predominant constituents, while fatty acids were more prevalent in zooxanthellate jellyfish [28,29,30].

### 3.2. Response Surface Models

The results of the hydrolysis time (X_1_) and alcalase, flavourzyme, and papain concentration (X_2_) on the jellyfish hydrolysate are shown in Table 1. A comprehensive response surface model was fitted using regression analysis. The regression coefficients for the full-2nd-order response surface models are shown in Table 2. Most of the models for each enzyme were significant (*p* ≤ 0.05) terms. The coefficients of determination (*R^2^*) were 0.78–0.99. Furthermore, the lack of fit for the models were not significant (*p* > 0.05), indicating relationships among the selected parameters as well as a high degree of confidence for explaining the effect of the variables in the observed data [31].

**Figure 1:**
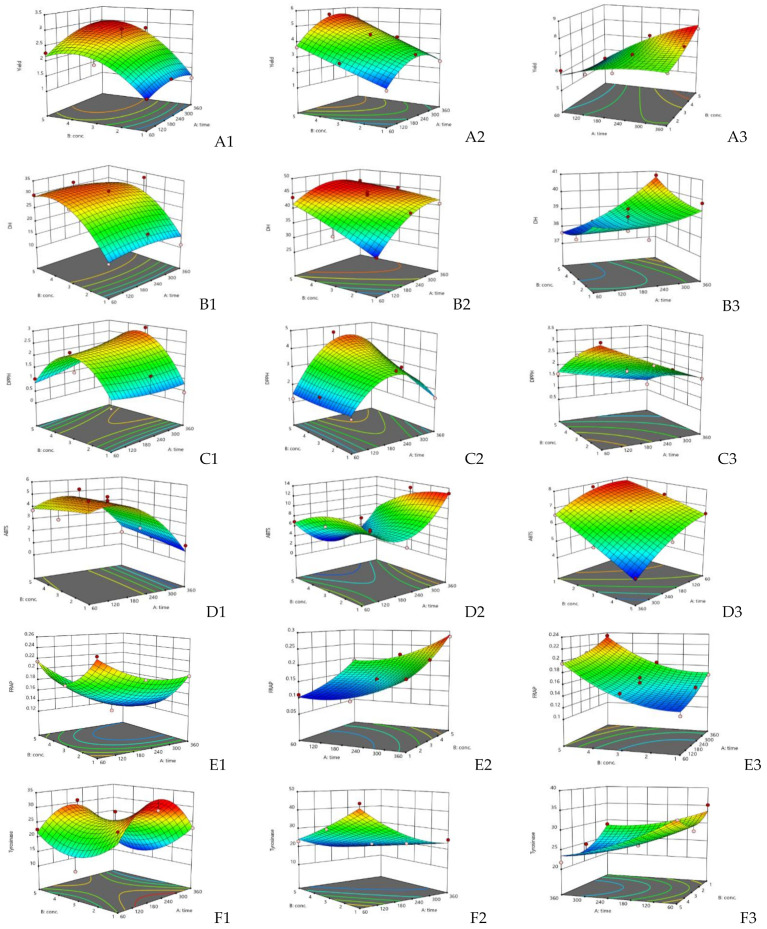
Response surface plots for DH, yield, IC_50_ of DPPH and ABTS radical scavenging, and tyrosinase inhibitory activity: and FRAP value using alcalase (**A1**–**F1**), flavourzyme (**A2**–**F2**), and papain (**A3**–**F3**) hydrolysis.

**Figure 2:**
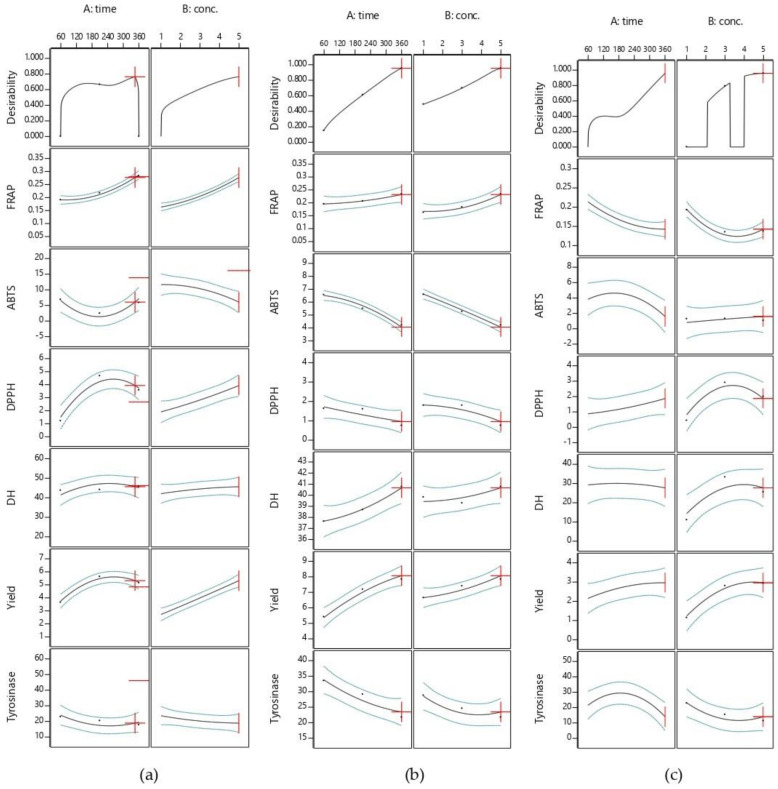
Plots of the main effects of hydrolysis time (X_1_) and enzyme concentration (X_2_) on DH, yield, DPPH and ABTS radical scavenging, FRAP value, and tyrosinase inhibition activity using alcalase (**a**), flavourzyme (**b**) and papain (**c**) hydrolysis.

**Figure 3:**
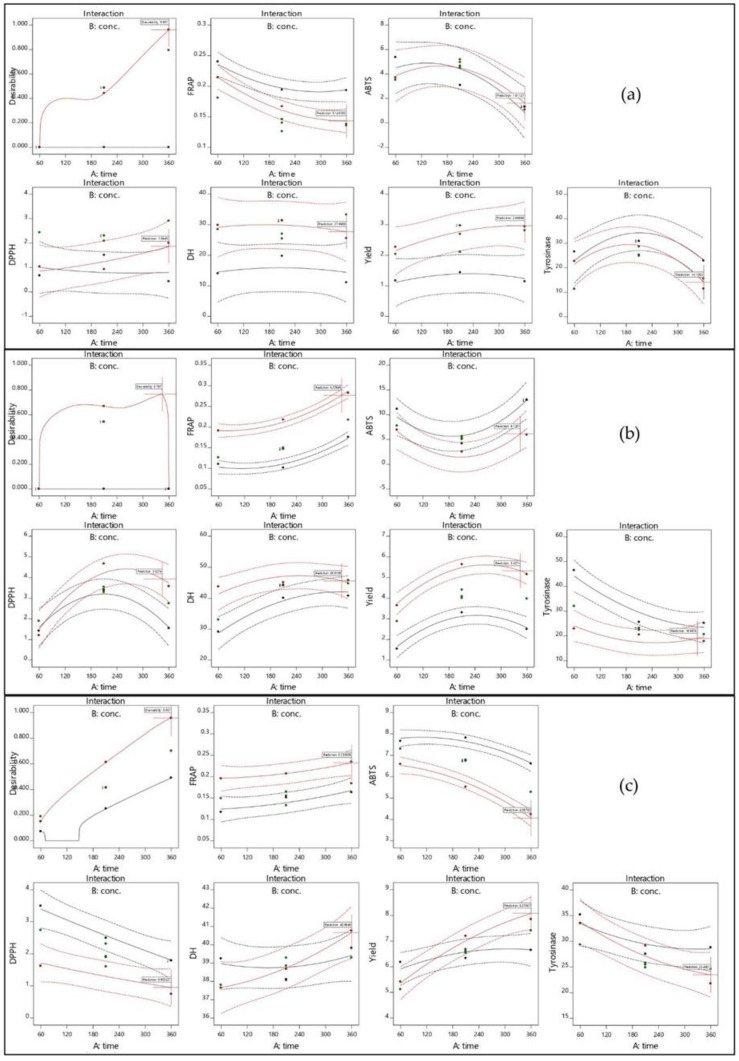
Plot of the interaction effect for hydrolysis time (X_1_) and enzyme concentration (X_2_) on DH, yield, DPPH and ABTS radical scavenging, FRAP value, and tyrosinase inhibition activity using alcalase (**a**), flavourzyme (**b**), and papain (**c**) hydrolysis.

The plots of the response surface that were used to predict the effects of the hydrolysis time and concentration of alcalase, flavourzyme, or papain on the responses are shown in three-dimensional plots in Figure 1. For flavourzyme, both the hydrolysis time and enzyme concentration had the great effect on yield (Figure 1A1–A3), while the enzyme concentration and hydrolysis time and that most significant effect on alcalase and papain, respectively (Figure 2). Both independent factors were found to impact on yield in papain (Figure 3). Protein hydrolysate yield increased as the hydrolysis time and enzyme concentration increased. This might be due to the higher amount of peptide cleavage in the native protein during enzyme hydrolysis and the conversion to shorter or longer peptides, resulting in a higher hydrolysate yield. Similar results were also obtained in previous studies for eel protein hydrolysate [32] and herring muscle [33]. Various factors may have influenced hydrolysate yield, but type of enzyme used for hydrolysis had the greatest effect on the yield and properties [34]. This study ranks the hydrolysis yields of alcalase, flavourzyme, and papain, as shown in Table 1. Flavourzyme is a combination of endo- and exopeptidases that can produce both free amino acids and peptides, while alcalase and papain are endopeptidases that can hydrolyze protein with a high degree of specificity, particularly peptide bonds, and have a preference for uncharged residues [35,36].

The effects of independent variables on the DH of JFH are shown in Figure 1B1–B3. The hydrolysis time had a significant effect on the DH for flavourzyme and papain, while the enzyme concentration had a significant effect on alcalase and flavourzyme (Figure 2). The DH increased as the enzyme concentration and hydrolysis time increased. The DH of jellyfish hydrolysate in this study was similar to those of previous studies, such as herring beluga viscera [37], tuna dark muscle by-products [17,38], and channel catfish skin [39]. Normally, the DH is higher when the reaction time is longer and increases rapidly at the beginning of a reaction before showing down and stabilizing [40,41]. The DH reflects the percentage of peptide bonds broken down by each protease. This allows the stage where number and size of peptides will have the best activity to be determined [40].

As shown in Figure 1C1–E3 exhibit the effects of the independent factors on the three responses to antioxidant activity: DPPH, ABTS, and FRAP, respectively. For flavourzyme and papain, the IC_50_ value of the DPPH radical scavenging activity was influenced by both independent variables. The duration of hydrolysis and the enzyme concentration demonstrated significant effects on the IC_50_ value of the ABTS radical scavenging activity of alcalase and flavourzyme hydrolysates, respectively, whereas both independent variables were caused significant effects in papain (Figure 2). However, the interaction between both independent variables affected the DPPH and ABTS for both flavourzyme and papain, respectively (Figure 3). Increasing the enzyme concentration and hydrolysis time increased the DH and led to good antioxidant activity in the jellyfish hydrolysates. The smaller peptides possessed more metal chelating and radical scavenging capabilities than the larger peptides, as per the results of Saidi et al. [41]. Different enzymes cleaved the peptide bonds at different positions in the protein structure, resulting in varying peptide sequences with various antioxidant activities [42]. Increasing the number of hydrophobic peptides showed a high reaction rate against free radicals [32]. After increasing the DH from 15 to 65%, a goby muscle hydrolysate short peptide with a stronger antioxidant was generated [43].

The effect of the independent variables on the IC_50_ value of the tyrosinase inhibition activity of jellyfish hydrolysate are shown in Figure 1F1–F3. Both the enzyme concentration and hydrolysis time had a significant effect on flavourzyme, while hydrolysis time significantly affected papain (Figure 2). The interaction effect for both independent variables on the tyrosinase inhibition activity was only in papain (Figure 3). Increased hydrolysis times and greater enzyme concentrations resulted in decreased IC_50_ values for tyrosinase inhibition activity (indicating strong tyrosinase inhibitory). Tyrosinase is a Cu^2+^-containing enzyme that plays an important role in melanogenesis and that could be catalyzed by quinones [44]. Therefore, antioxidative substances with the ability to prohibit the oxidative pathway and/or binding to the tyrosinase activity site (Cu^2+^) could inhibit the catalytic reaction of the enzymes. Polar or uncharged amino acid residues such as Ser and Cys are typically found in effective tyrosinase inhibitor peptides [4]. According to the report by Masuda et al. [45], some tyrosinase inhibitors also have strong antioxidant activity.

### 3.3. Multiple Response Optimization and Model Validation

The desirability function of the Design Expert Statistical program was used to optimize the hydrolysis conditions using multiple response optimization. Maximum targets were established for the DH, yield, and FRAP values, whereas minimum goals were set for the IC_50_ values for DPPH, ABTS radical scavenging, and tyrosinase inhibition activity. Table 3 shows the individual and composite desirability values of each response, assuming that all of the responses were evaluated equally. The results indicated that the optimal conditions for each enzyme were an enzyme concentration of 5% and hydrolysis times of 360, 345, or 360 min for alcalase (JFAH), flavourzyme (JFFH), and papain (JFPH), respectively. These optimal conditions were validated to obtain the experimental values. As indicated in Table 3, the predicted values were not substantially different from the experiment values (*p* > 0.05). The results indicated that they were good predictors. Based on the experimental values, some of which show better bioactivity than others, the most effective was JFFH, which was chosen for further research.

### 3.4. Amino Acid Profile

JFFH’s amino acid profile presented in Table 4. Gly, Ala, Glx, Alx, and Pro were the most abundant amino acids in JFFH, accounting for around 18, 14, 13, 9, and 9% of the total amino acid composition, respectively. The hydrophobic amino acids Ala, Tyr, Val, Met, Cys, Ile, Phe, Trp, Leu, and Pro composed 43% of JFFH, whereas the other ~57% of JFFH comprised hydrophilic amino acids. The hydrophobic amino acid groups are important for bioactivity, especially in terms of antioxidant and antiproliferative activity of protein hydrolysates [46,47]. As a consequence, JFFH’s stronger antioxidant and tyrosinase inhibition activity might be related to its higher hydrophobic amino acid content. According to Schurink et al. [4], peptides possessing polar, uncharged amino acid residues, such as Ser and Cys, are similarly effective in inhibiting tyrosinase. The tyrosine-inhibitory activity might be related to a thiol group in a Cys-containing peptide chelating copper ions [48].

### 3.5. Effect of Jellyfish Hydrolysate on Cell Viability

**Figure 4:**
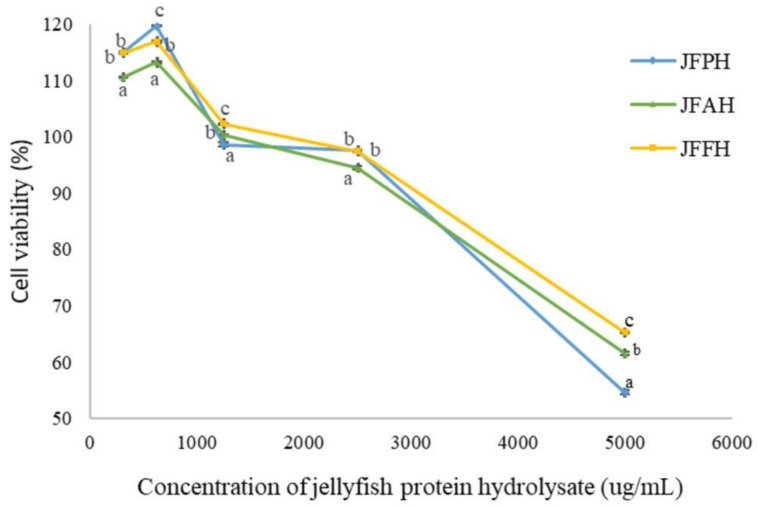
Cytotoxic effect of jellyfish hydrolysate prepared with alcalase (JFAH), flavourzyme (JFFH), and papain (JFPH) on human keratinocytes cells (HaCaT) at 24 h incubation.

The effect on cell viability of jellyfish hydrolysate was evaluated based on the MTT value in the HaCaT cell lines, as shown in Figure 4. The cell viability was calculated compared to that of HaCaT that had been cell-cultured without jellyfish hydrolysate (control), reporting an 80% (IC_80_ value) survival rate. This result showed that the cell survival increased when the concentration increased from 0.313 to 0.625 mg/mL for all hydrolysates and decreased afterwards. The JFPH, JFAH, and JFFH concentrations of 0.313–1.25 mg/mL resulted in cell viability >100% as well as low cytotoxicity and potentially enhanced cell growth. Even at the highest dose (5 mg/mL), the jellyfish hydrolysate was non-cytotoxic. Domenico et al. [20] reported that the crude aqueous soluble protein (SP) of *R*. *pulmo* was able to diminish the cell viability of human epidermal keratinocyte to 60% when the SP was added >2.5 µg/mL. They also discovered that the fractioned SP with MW >30 kDa was cytotoxic at concentration doses as low as 1 mg/mL, while SP fractions with MW concentrations of 10–30 kDa and 3–10 kDa were non-cytotoxic, even at the maximum dosage (10 µg/mL). Human breast cancer cells (MCF-7), human hepatocellular carcinoma cells (HepG2), human normal fibroblasts (HFB4), breast cancer cells (MDA-MB231), and hepatocellular carcinoma (HCC) all demonstrated that the dose-dependent reduction in cell viability was dependent on the concentration of the extracts and the duration of the contact time [49,50]. The IC_80_ value of JFFH, JFAH, and JFPH was 2.5 µg/mL. Therefore, jellyfish hydrolysate demonstrated low toxicity to HaCaT at this concentration. However, further research is still required in order to assess the potential of jellyfish hydrolysate as functional ingredient in different applications.

### 3.6. Fractionation of JFFH

The JFFH was fractionated into four fractions: fraction-I (>10 kDa), fraction-II (3–10 kDa), fraction-III (1–3 kDa), and fraction-IV (<1 kDa), to identify which fractions exhibit functional properties. Table 5 showed the yield, antioxidant properties, and tyrosinase inhibition activity for each fraction. Fraction-III had the highest antioxidant activity and tyrosinase inhibition activity (*p* ≤ 0.05). These results are consistent with those determined by Mongkonkamthorn et al. [17] and Mongkonkamthorn et al. [19], who demonstrated that peptides with <1 kDa MW from tuna blood and dark meat had the strongest antioxidant activity. Zhuang et al. [12] reported jellyfish hydrolysate fraction HF-2 (1–3 kDa) has a tyrosinase inhibitory effect that is higher than 50% at 5 mg/mL as well as strong Cu^2+^ chelating activity. Prakot et al. [51] also reported a possible tyrosinase inhibitor in spotted babylon protein hydrolysate (MW < 5 kDa), reporting an IC_50_ value of 0.075 µg/mL.

### 3.7. Gel Filtration Chromatography

**Figure 5:**
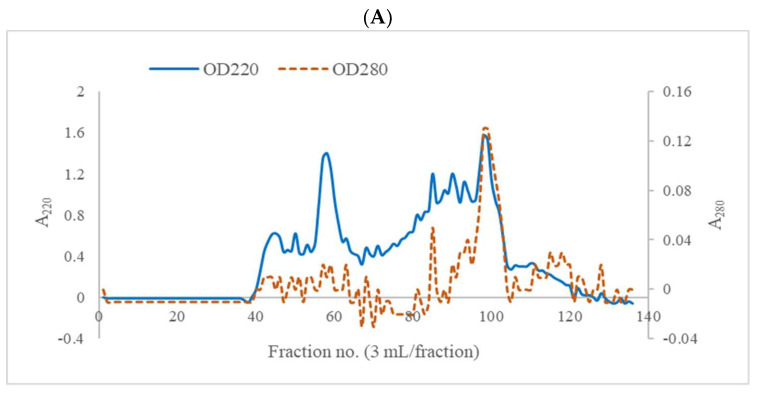

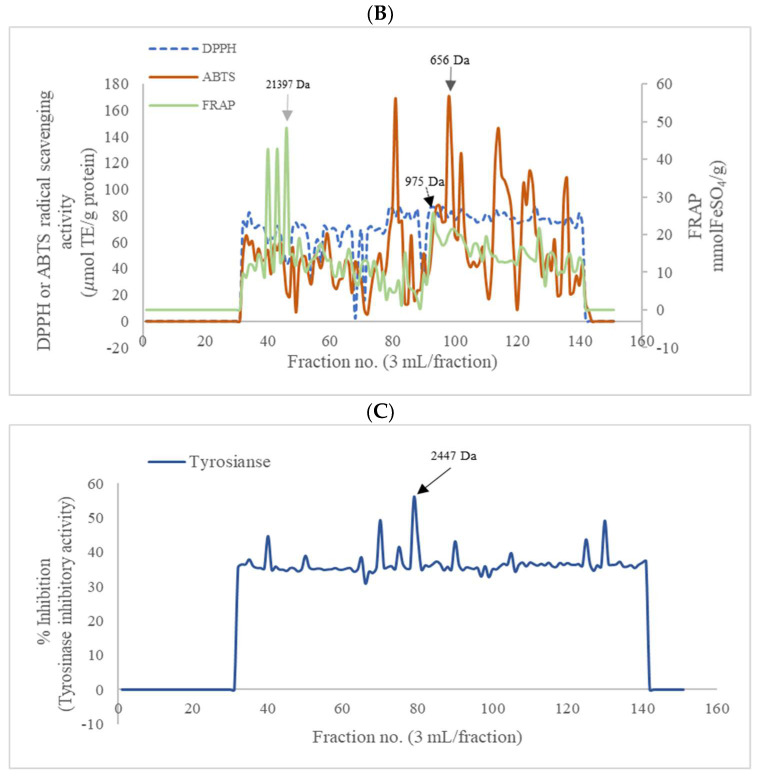
Elution profile of antioxidant and tyrosinase inhibitory peptide from fraction-III (MW 1–3 kDa) from JFFH using Sephadex G-25 gel filtration chromatography monitored at A_220_ and A_280_ (**A**). DPPH and ABTS radical scavenging and FRAP value (**B**) and tyrosinase inhibitory activity (**C**) of the different fractions.

As represented in Figure 5A, the fraction-III of JFFH was further isolated using a Sephadex G-25 column. The peptide bonds and the proteins, peptides, or amino acids with aromatic rings were able to be monitored using A_220_ and A_280_, respectively [52]. Many individual peaks were observed at A_280_, indicating the presence of aromatic peptide or amino acid side chains. Fraction-III showed the same distinct peaks at A_220_ and A_280_ in fraction no. 97, which may indicate the presence peptide linkages and that aromatic amino acids rings are abundant (tyrosine and tryptophan). The DPPH and ABTS radical scavenging activity as well as the FRAP value (Figure 5B) and the tyrosinase inhibitory activity of the fractions (Figure 5C) were all investigated. Different fractions showed varying degrees of antioxidant activity and tyrosinase inhibition. Fraction no. 42, which exhibited the highest FRAP activity, contained peptides with an MW of 21,397 Da. The highest DPPH radical scavenging activity was shown in fraction no. 95 (peptide with MW of 807 Da). The strongest ABTS radical scavenging activity was in the compressed peptides with an MW of 656 Da. According to Wu et al. [53], antioxidant peptides from mackerel protein hydrolysate with a molecular weight of 1400 Da had higher activity than peptides with molecular weights of 900 Da or 200 Da. Mendis et al. [54] observed the strongest antioxidant activity in peptides from giant squid skin gelatin hydrolysate with an MW less than 3000 Da. The isolated peptides from short-sequence and low-MW (538–887 Da) tuna by-product hydrolysate showed strong antioxidant properties that affected the radical values and lipid oxidation inhibition [28]. Therefore, fraction-III, which was isolated from JFFH-containing peptides with an MW of 2448 Da, showed the strongest tyrosinase inhibitory activity. The hydrophobic Val, Ala, and/or Leu residues in potent tyrosinase inhibitory peptides are generally combined with Arg and/or Phe [4]. Sea cucumber (*B. Vitiensis*) demonstrated good tyrosinase inhibitory activity, with an IC_50_ value of 0.28 mg/mL [55]. Many studies have revealed that the amino acid composition and molecular weight distribution of certain peptides were associated with their antioxidant activity and strong metal chelation capacity [4,12,21].

## 4. Conclusions

The optimized conditions for the production of jellyfish hydrolysate using alcalase, flavourzyme, and papain were an enzyme concentration of 5%, with hydrolysis times of 360 min for alcalase, 345 min for flavourzyme, and 360 min for papain. The models that were generated fit the experimental values well. The jellyfish hydrolysate obtained flavourzyme hydrolysis (JFFH) contained hydrophobic and hydrophilic amino acids, which comprised about about 43 and 57% of the total amino acids, respectively. The IC_80_ value for HaCaT cell viability for JFAH, JFFH, and JFPH was 2.5 mg/mL. The fractionated peptides from JFFH fraction-III (MW of 1–3 kDa) had highest antioxidant activity and strongest tyrosinase inhibition activity. The peptides in JFFH with high ABTS radical scavenging, DPPH radical scavenging, and FRAP values were characterized as having MWs of 656, 807 and 21,397 Da, respectively, while the fractionated peptide with an MW of 2448 Da showed high tyrosinase inhibition activity. Therefore, it is possible to utilize salted jellyfish as a raw material for the preparation of natural antioxidants and as an anti-tyrosinase, meaning that it can potentially be used as functional ingredient in the food, cosmetic, and pharmaceutical industries. 

## Figures and Tables

**Table 1:** Experimental units and response variables for DH, yield, DPPH and ABTS radical scavenging activity, FRAP value, and tyrosinase inhibitory activity of jellyfish protein hydrolyzed using alcalase, flavourzyme, and papain.

**Enzyme**	**Treatment**	**Factors**		**Responses**					
		**X_1_**	**X_2_**	**%DH**	**%Yield**	**DPPH (IC_50_)** **(mg** **/mL)**	**ABTS (IC_50_)** **(mg** **/mL)**	**FRAP** **(mmol FeSO_4_** **/g)**	**Anti** **-Tyrosinase (IC_50_)** **(mg** **/mL)**
Alcalase	1	60	1	14.0 ^b^ ± 0.2	1.17 ^a^ ± 0.23	0.54 ^a^ ± 0.26	5.23 ^g^ ± 0.20	0.24 ^h^ ± 0.03	23.2 ^d^ ± 0.6
	2	360	1	11.1 ^a^ ± 0.2	1.12 ^a^ ± 0.23	0.42 ^a^ ± 0.07	6.03 ^gh^ ± 1.69	0.19 ^e^ ± 0.03	22.9 ^c^ ± 0.5
	3	60	5	29.8 ^g^ ± 0.1	2.27 ^d^ ± 0.52	1.02 ^b^ ± 0.17	3.70 ^def^ ± 0.92	0.21 ^f^ ± 0.02	26.0 ^de^ ± 0.7
	4	360	5	25.5 ^e^ ± 0.1	2.93 ^f^ ± 0.80	2.15 ^cd^ ± 0.43	1.07 ^a^ ± 0.02	0.13 ^a^ ± 0.01	11.4 ^a^ ± 0.1
	5	60	3	28.4 ^f^ ± 0.1	2.03 ^d^ ± 0.32	2.35 ^d^ ± 0.18	3.17 ^de^ ± 0.57	0.22 ^g^ ± 0.07	13.7 ^a^ ± 1.0
	6	360	3	33.2 ^i^ ± 1.7	2.81 ^f^ ± 0.84	7.70 ^e^ ± 2.47	1.43 ^b^ ± 0.17	0.13 ^a^ ± 0.01	25.5 ^e^ ± 0.2
	7	210	1	19.7 ^c^ ± 0.1	1.43 ^b^ ± 0.12	1.50 ^bc^ ± 1.18	2.02 ^bc^ ± 1.63	0.19 ^e^ ± 0.03	30.8 ^g^ ± 0.7
	8	210	5	31.4 ^h^ ± 0.1	2.69 ^ef^ ± 0.46	0.92 ^ab^ ± 0.43	4.02 ^ef^ ± 1.04	0.17 ^c^ ± 0.02	28.9 ^fg^ ± 1.9
	9	210	3	28.2 ^f^ ± 0.1	2.97 ^f^ ± 0.11	2.40 ^d^ ± 0.36	4.63 ^f^ ± 0.23	0.14 ^b^ ± 0.01	23.9 ^d^ ± 0.1
	10	210	3	24.1 ^d^ ± 0.1	2.97 ^f^ ± 0.18	2.14 ^cd^ ± 0.27	5.21 ^g^ ± 0.40	0.13 ^a^ ± 0.01	25.7 ^de^ ± 1.8
	11	210	3	31.3 ^h^ ± 0.1	1.76 ^bc^ ± 0.32	1.14 ^b^ ± 0.08	2.97 ^c^ ± 0.30	0.18 ^d^ ± 0.02	20.5 ^b^ ± 0.1
Flavourzyme	1	60	1	29.1 ^a^ ± 0.1	1.55 ^a^ ± 0.03	1.16 ^bc^ ± 0.28	11.1 ^ef^ ± 2.8	0.14 ^d^ ± 0.01	43.8 ^j^ ± 0.1
	2	360	1	40.8 ^c^ ± 0.0	2.50 ^b^ ± 0.11	0.63 ^a^ ± 0.07	20.6 ^g^ ± 1.2	0.14 ^d^ ± 0.01	22.7 ^bc^ ± 0.5
	3	60	5	42.7 ^d^ ± 0.1	3.65 ^ef^ ± 0.24	2.22 ^e^ ± 0.88	9.76 ^e^ ± 0.91	0.13 ^c^ ± 0.01	29.4 ^f^ ± 0.5
	4	360	5	44.3 ^f^ ± 0.1	5.15 ^g^ ± 0.28	0.86 ^b^ ± 0.07	5.91 ^bc^ ± 0.14	0.14 ^d^ ± 0.01	13.5 ^cd^ ± 0.5
	5	60	3	43.0 ^e^ ± 0.0	2.88 ^b^ ± 0.14	1.75 ^d^ ± 0.24	7.74 ^d^ ± 0.88	0.14 ^d^ ± 0.01	30.1 ^gh^ ± 1.4
	6	360	3	45.7 ^g^ ± 0.3	3.97 ^f^ ± 0.15	2.57 ^ef^ ± 1.24	12.9 ^f^ ± 0.7	0.13 ^c^ ± 0.01	23.2 ^c^ ± 1.3
	7	210	1	43.1 ^e^ ± 0.1	3.30 ^c^ ± 0.12	2.41 ^ef^ ± 1.34	4.15 ^b^ ± 1.11	0.17 ^f^ ± 0.05	35.8 ^i^ ± 0.4
	8	210	5	38.1 ^b^ ± 0.1	5.63 ^g^ ± 1.18	4.66 ^h^ ± 0.17	2.49 ^a^ ± 0.31	0.15 ^e^ ± 0.02	33.5 ^h^ ± 0.2
	9	210	3	45.3 ^g^ ± 0.1	3.40 ^d^ ± 0.20	1.50 ^d^ ± 0.08	5.26 ^b^ ± 1.68	0.12 ^b^ ± 0.02	22.5 ^b^ ± 0.2
	10	210	3	44.9 ^f^ ± 0.1	3.57 ^e^ ± 0.12	2.71 ^f^ ± 0.41	6.03 ^c^ ± 0.14	0.11 ^a^ ± 0.02	20.7 ^a^ ± 0.7
	11	210	3	42.2 ^de^ ± 0.1	5.02 ^g^ ± 0.39	2.79 ^f^ ± 0.52	2.51 ^a^ ± 0.08	0.16 ^ef^ ± 0.06	24.4 ^e^ ± 0.8
Papain	1	60	1	36.1 ^a^ ± 0.1	4.29 ^b^ ± 0.11	1.74 ^c^ ± 0.89	6.81 ^ef^ ± 0.47	0.14 ^a^ ± 0.00	46.2 ^h^ ± 1.5
	2	360	1	37.1 ^b^ ± 0.1	5.41 ^e^ ± 0.09	1.60 ^bc^ ± 1.25	8.27 ^efg^ ± 1.10	0.15 ^b^ ± 0.01	29.4 ^f^ ± 0.4
	3	60	5	38.8 ^e^ ± 0.1	3.79 ^b^ ± 0.70	1.30 ^ab^ ± 0.57	3.42 ^b^ ± 0.16	0.16 ^c^ ± 0.07	31.8 ^g^ ± 0.1
	4	360	5	38.7 ^d^ ± 0.1	7.36 ^ij^ ± 0.40	0.89 ^a^ ± 0.26	5.12 ^c^ ± 0.77	0.16 ^c^ ± 0.01	24.2 ^d^ ± 0.7
	5	60	3	39.9 ^fg^ ± 0.0	2.70 ^a^ ± 0.92	0.87 ^a^ ± 0.24	2.65 ^a^ ± 0.01	0.13 ^a^ ± 0.01	28.4 ^e^ ± 0.1
	6	360	3	40.4 ^g^ ± 0.2	6.63 ^hi^ ± 0.44	1.79 ^cd^ ± 0.88	5.53 ^d^ ± 0.08	0.20 ^e^ ± 0.02	18.5 ^ab^ ± 0.5
	7	210	1	42.7 ^h^ ± 0.1	5.50 ^ef^ ± 0.75	1.40 ^b^ ± 0.20	5.28 ^cd^ ± 0.29	0.17 ^d^ ± 0.02	32.0 ^g^ ± 1.9
	8	210	5	37.8 ^c^ ± 0.1	7.91 ^j^ ± 0.16	1.95 ^d^ ± 1.30	5.03 ^c^ ± 0.25	0.20 ^e^ ± 0.02	22.5 ^c^ ± 1.0
	9	210	3	39.8 ^fg^ ± 0.1	6.12 ^fg^ ± 0.45	2.81 ^e^ ± 0.89	6.80 ^ef^ ± 0.46	0.17 ^d^ ± 0.01	17.5 ^a^ ± 0.9
	10	210	3	37.9 ^c^ ± 0.1	6.25 ^gh^ ± 0.23	2.94 ^f^ ± 0.41	6.70 ^ef^ ± 0.40	0.17 ^d^ ± 0.02	19.2 ^b^ ± 1.5
	11	210	3	39.6 ^f^ ± 0.2	7.05 ^i^ ± 0.35	1.99 ^d^ ± 0.32	6.67 ^e^ ± 0.57	0.16 ^c^ ± 0.01	19.5 ^b^ ± 0.2

Note: Mean ± SD, X_1_: hydrolysis time (min), X_2_: enzyme concentration (%); different letters in the same column of each enzyme show significant differences (*p* ≤ 0.05).

**Table 2:** Full quadratic model of jellyfish hydrolysis using alcalase, flavourzyme, and papain.

Enzyme	Responses	Quadratic Polynomial Model	*R* ^2^	*p*-Value
Alcalase	DPPH (IC_50_) (mg/mL)	Y_1_ = −0.429 − 0.004X_1_ + 1.912X_2_ + 0.001X_1_X_2_ + 0.00001X_1_^2^ − 0.335X_2_^2^	0.8393	0.0469
	ABTS (IC_50_) (mg/mL)	Y_2_ = 3.74 + 0.019X_1_ − 0.172X_2_ − 0.0001X_1_^2^ − 0.010X_2_^2^ + 0.001X_1_X_2_	0.8377	0.0479
	FRAP (mmol FeSO_4_/g)	Y_3_ = 0.316 − 0.001X_1_ − 0.065X_2_ − 0.00003X_1_X_2_ + 0.000001X_1_^2^ + 0.010X_2_^2^	0.9729	0.0006
	%DH	Y_4_ = 0.93 + 0.028X_1_ + 13.9X_2_ − 0.0001X_1_^2^ − 1.69X_2_^2^ − 0.001X_1_X_2_	0.8282	0.0547
	%Yield	Y_5_ = −0.071 + 0.004X_1_ + 1.09X_2_ − 0.00001X_1_^2^ –0.145X_2_^2^ + 0.001X_1_X_2_	0.886	0.021
	Tyrosinase (IC_50_) (mg/mL)	Y_6_ = 19.6 + 0.218X_1_ − 9.14X_2_ − 0.001X_1_^2^ + 1.53X_2_^2^ − 0.01X_1_X_2_	0.8433	0.0442
Flavourzyme	DPPH (IC_50_) (mg/mL)	Y_1_ = 0.29 + 0.028X_1_ − 0.287X_2_ −0.0001X_1_^2^ + 0.03X_2_^2^ + 0.002X_1_X_2_	0.9367	0.0051
	ABTS (IC_50_) (mg)	Y_2_ = 12.9 –0.09X_1_ + 1.67X_2_ + 0.0003X_1_^2^ − 0.37X_2_^2^ − 0.002X_1_X_2_	0.8939	0.0177
	FRAP (mmol FeSO_4_/g)	Y_3_ = 0.15 + 0.04X_1_ + 0.05X_2_ + 0.003X_1_^2^ + 0.02X_2_^2^ + 0.01X_1_X_2_	0.9913	<0.0001
	%DH	Y_4_ = 18.0 + 0.12X_1_ +4.60X_2_ − 0.002X_1_^2^ − 0.16X_2_^2^ − 0.01X_1_X_2_	0.9091	0.0122
	%Yield	Y_5_ = 0.05 + 0.02X_1_ + 0.45X_2_ − 0.0001X_1_^2^ + 0.01X_2_^2^ + 0.01X_1_X_2_	0.9801	0.0003
	Tyrosinase (IC_50_) (mg/mL)	Y_6_ = 59.6 − 0.16X_1_ − 7.39X_2_ + 0.002X_1_^2^ + 0.26X_2_^2^ + 0.014X_1_X_2_	0.9375	0.005
Papain	DPPH (IC_50_) (mg)	Y_1_ = 3.98 – 0.01X_1_ – 0.19X_2_ + 0.000002X_1_^2^ – 0.05X_2_^2^ + 0.001X_1_X_2_	0.9341	0.0056
	ABTS (IC_50_) (mg/mL)	Y_2_ = 7.80 + 0.01X_1_ – 0.17X_2_ – 0.00002X_1_^2^ – 0.013X_2_^2^ –0.001X_1_X_2_	0.9874	<0.0001
	FRAP (mmol FeSO_4_/g)	Y_3_ = 0.13 + 0.00002X_1_ – 0.01X_2_ + 0.0000003X_1_^2^ + 0.01X_2_^2^ – 0.00001X_1_X_2_	0.9253	0.0076
	%DH	Y_4_ = 40.1 – 0.01X_1_ – 0.87X_2_ + 0.00002X_1_^2^ + 0.07X_2_^2^ + 0.002X_1_X_2_	0.7832	0.0924
	% Yield	Y_5_ = 5.99 + 0.01X_1_ – 0.55X_2_ – 0.00001X_1_^2^ + 0.05X_2_^2^ + 0.002X_1_X_2_	0.9388	0.0047
	Tyrosinase (IC_50_) (mg/mL)	Y_6_ = 39.2 – 0.04X_1_ – 4.01X_2_ + 0.0001X_1_^2^ + 0.73X_2_^2^ – 0.01X_1_X_2_	0.8818	0.0229

Note: Mean ± SD, X_1_: hydrolysis time (min), X_2_: enzyme concentration (%).

**Table 3:** Optimized hydrolysis conditions and verification model for all responses of jellyfish hydrolysate using alcalase (JFAH), flavourzyme (JFFH), and papain (JFPH) hydrolysis.

Enzyme	Value	Factors		Responses					
		X_1_ (min)	X_2_ (%)	%DH	%Yield	DPPH (IC_50_) (mg/mL)	ABTS (IC_50_) (mg/mL)	FRAP (IC_50_) (mmol FeSO_4_/g)	Tyrosinase Inhibitory (IC_50_) (mg/mL)
JFAH	Predicated value			27.7	2.97	2.30	1.61	0.14	14.1
	Experimental value	360	5	28.2 ^a^ ± 1.1	3.01 ^a^ ± 0.04	2.5 ^c^ ± 0.1	1.81 ^a^ ± 0.02	0.13 ^a^ ± 0.05	14.9 ^b^ ± 0.0
	Composite desirability	0.96							
JFFH	Predicated value			45.6	5.32	0.81	5.40	0.17	19.0
	Experimental value	345	5	47.3 ^c^ ± 0.3	6.40 ^b^ ± 0.03	0.73 ^a^ ± 0.14	2.58 ^b^ ± 0.19	0.23 ^b^ ± 0.04	14.1 ^ab^ ± 0.1
	Composite desirability	0.96							
JFPH	Predicated value			40.7	7.45	0.95	4.30	0.23	23.5
	Experimental value	360	5	41.8 ^b^ ± 0.5	7.21 ^bc^ ± 1.05	0.98 ^b^ ± 0.04	4.50 ^c^ ± 0.08	0.27 ^c^ ± 0.02	24.5 ^c^ ± 0.0
	Composite desirability	0.95							

Note: Mean ± SD, X_1_: hydrolysis time (min), X_2_: enzyme concentration (%); different superscripts in the same experimental values of response show significant differences (*p* ≤ 0.05).

**Table 4:** Amino acid content in jellyfish hydrolysate using flavourzyme hydrolysis (JFFH).

Amino Acid	g Amino Acid/100 g of Sample
Asn + Asp	5.00
Gln + Glu	6.96
Ser	1.85
Thr	1.46
His	0.23
Gly	9.74
Ala	4.50
Arg	3.53
Tyr	0.60
Val	1.62
Met	0.45
Cys	7.70
Ile	1.23
Phe	0.48
Trp	0.19
Leu	2.02
Lys	2.27
Pro	4.97
Total amino acid	54.76

**Table 5:** Antioxidant and tyrosinase inhibition activity and yield from the fractionation of jellyfish hydrolysates using flavourzyme.

MW (kDa)		Responses				
	Fraction	ABTS (IC_50_) (mg/mL)	DPPH (IC_50_) (mg/mL)	FRAP (mmol FeSO_4_/g)	Tyrosinase Inhibitory (IC_50_) (mg/mL)	Yield (%)
>10 kDa	I	2.91 ^c^ ± 0.01	3.71 ^c^ ± 0.11	0.65 ^c^ ± 0.001	14.2 ^b^ ± 8.49	3.01 ^d^ ± 0.01
10–3 kDa	II	1.15 ^b^ ± 0.01	0.85 ^a^ ± 0.03	0.27 ^b^ ± 0.001	9.35 ^a^ ± 0.27	2.18 ^c^ ± 0.02
3–1 kDa	III	0.91 ^a^ ± 0.01	0.95 ^a^ ± 0.04	0.24 ^a^ ± 0.001	8.95 ^a^ ± 0.01	1.89 ^b^ ± 0.00
< 1 kDa	IV	0.89 ^a^ ± 0.01	1.11 ^b^ ± 0.01	0.28 ^b^ ± 0.01	12.6 ^b^ ± 0.14	0.73 ^a^ ± 0.00

Note: Mean ± SD, DPPH and ABTS radical scavenging activity, FRAP value and tyrosinase inhibitory activity; different letters in the same variables show significant differences (*p* ≤ 0.05).

## Data Availability

Data is contained within the article.
